# Genetic diversity and agro-morphological characterization of cassava varieties provides insight for breeding and crop improvement

**DOI:** 10.1038/s41598-025-02527-5

**Published:** 2025-05-20

**Authors:** Shamal Shasang Kumar, Shalendra Prasad, Owais Ali Wani, Salah El-Hendawy, Mohamed A. Mattar, Ali Salem

**Affiliations:** 1 Crop Research Division, Ministry of Agriculture & Waterways (MOA & W), P.O. Box 77, Nausori, Fiji; 2 Division of Agronomy, Faculty of Agriculture, Sher-e-Kashmir University of Agricultural Sciences and Technology of Kashmir, Wadura, Sopore, 193201 India; 3 Department of Plant Production, College of Food and Agricultural Sciences, King Saud University, P.O. Box 2460, 11451 Riyadh, Saudi Arabia; 4 Prince Sultan Bin Abdulaziz International Prize for Water Chair, Prince Sultan Institute for Environmental, Water and Desert Research, King Saud University, P.O. Box 2454 Riyadh 11451, Saudi Arabia; 5 Department of Agricultural Engineering, College of Food and Agriculture Sciences, King Saud University, P.O. Box 2460 Riyadh 11451, Saudi Arabia; 6 Civil Engineering Department, Faculty of Engineering, Minia University, Minia 61111, Egypt; 7 Structural Diagnostics and Analysis Research Group, Faculty of Engineering and Information Technology, University of Pécs, Pécs 7622, Hungary

**Keywords:** Genetic variability, Cassava varieties, Agro-morphological traits, Principal component analysis, Correlation analysis, Hierarchical clustering, Breeding programs, Agroecology, Agroecology

## Abstract

The lack of knowledge about genetic variation in cassava is a problem for Fiji’s efforts to improve its genetics. Using agro-morphological features, this study aimed to assess the genetic diversity and interrelationships among 33 cassava cultivars. A field investigation was conducted at the Dobuilevu Research Station using a randomized complete block design. Morphological analysis, based on qualitative and quantitative characteristics, divided the germplasm into three groups. In both the qualitative and quantitative trait datasets, two principal components were found to account for 36.31% and 43.45% of the total genetic variance, respectively. Qualitative features, such as branching habit and stem cortex color *(r = 0.19)*, petiole color and root cortex color *(r = 0.32)*, and leaf color and root shape *(r = 0.40)* were shown to have significant positive correlations. Similarly, quantitative parameters like starch content *(r = 0.25)* and the number of leaf lobes with yield *(r = 0.17)* showed significant associations. Based on morphological and genetic similarities, hierarchical clustering grouped the cultivars into three qualitative and five quantitative clusters. While the quantitative traits emphasized variability in yield, starch content, and iron content. The qualitative traits’ descriptive statistics revealed diverse phenotypic expressions, with dark green leaf color and cylindrical root form being the most common. These results demonstrate significant genetic variation across cassava cultivars, which can be used for genetic improvement initiatives, germplasm conservation, and short-term varietal release programs. To facilitate the development of more resilient and productive cassava cultivars, targeted breeding efforts aimed at improving yield, quality, and stress tolerance are recommended based on the significant phenotypic and genetic variation observed.

## Introduction

Cassava (*Manihot esculenta* Crantz) is a major tuber crop widely grown in tropical and subtropical regions. It serves as a staple food and a key source of income and sustenance for over 800 million people worldwide^1^. It is known by various names such as tapioca, mandioca, manioc, and yuca across different regions. Cassava is a perennial shrub belonging to the *Euphorbiaceae* family, specifically the *Crotonoideae* subfamily and the *Manihotae* tribe^2^. It is extensively cultivated for its starchy tubers, which make it a highly versatile crop used for human consumption, animal feed, and as a raw material in various industries across Africa, Asia, and Latin America^3^. Renowned for its high carbohydrate content and ease of cultivation, cassava serves as a vital cash crop supporting livelihoods, particularly in Fiji^4^.

In Fiji, cassava holds a special place in agriculture. It serves as a key contributor to food security, cultural heritage, nutrition, and rural livelihoods. Its adaptability to diverse climatic conditions, tolerance to poor soils, and resilience to drought have made it an indispensable crop. Cassava is one of the most widely consumed staple foods in Fiji, with around 59.2% of the population including it in their daily diet^5^. Over a decade, cassava in Fiji has gained economic importance beyond its traditional role. It remains a staple for subsistence farmers while also becoming a key export commodity, contributing significantly to local and international markets^6^. Cassava production has significantly increased in Fiji, with gross production volumes rising from 68,314.6 metric tonnes (mt) in 2017 to 86,194.5mt in 2021, and achieving a gross value of $48.0 million FJD in 2021. This growth is attributed partly to increased export demand and value addition^7^.

Despite its significance, cassava production in Fiji faces challenges such as declining soil fertility, climate change, pest and disease, and limited genetic improvement^8^. Understanding the agro-morphological diversity of cassava is fundamental for addressing these challenges, as it provides insights into the genetic variability, adaptability, and performance of cassava under local conditions. A major area of focus for population geneticists and conservation biologists involves determining the degree and extent of genetic variation. A thorough understanding of a crop’s genetic diversity is crucial for breeders when selecting desirable parent plants for their breeding programs. The characterization and evaluation of diversity are vital for effectively utilizing unique accessions in crop improvement and ensuring better conservation of genetic resources. Crossing diverse genotypes or accessions can lead to the development of high-yielding hybrids that are resistant to a range of abiotic and biotic stresses. One approach to characterizing and classifying cassava germplasm is through the use of agro-morphological descriptors.

Agro-morphological characterization systematically examines cassava’s physical and agronomic features, including leaf color, plant height, stem width, root shape, branching pattern, and yield potential. By examining key plant parts like leaves, stems, and roots, morphological descriptors help clearly differentiate between different cassava varieties. These traits are critical for identifying superior genotypes that can thrive in Fiji’s specific agro-ecological zones and for developing improved varieties through breeding programs. Moreover, this process enables the identification of landraces and traditional varieties that hold unique traits, which may be valuable for enhancing cassava’s resilience to environmental stresses. Several guides for cassava characterization exist, including the International Board for Plant Genetic Resources (IBPGR)^9^, which defines a set of relatively stable morphological traits for the characterization of cassava genotypes. Additionally, the International Institute of Tropical Agriculture (IITA)^10^ provides a list of selected morphological traits that are useful for cassava characterization. Morphological characterization has been used to analyze cassava accessions in various studies^11–14^ evaluated 53 cassava accessions using 29 different morphological traits and found that the accessions displayed relatively high genetic variation in both quantitative and qualitative characteristics^15^ assessed 43 cassava genotypes to evaluate genetic variability in root yield and its components, finding significant variation in plant height, root number, root weight, and dry matter among the genotypes^16^ evaluated seven cassava cultivars using 20 morphological traits and revealed that these cultivars could be identified using common descriptors such as the color in apical leaves, shape of the central leaflet, petiole orientation, foliar scars and the color of the stem epidermis.

There is very limited information available on the morphological characterization of cassava varieties in Fiji. Therefore, this study aimed to characterize the agro-morphological traits of cassava varieties in Fiji. The findings will provide essential information for cassava improvement initiatives, promote sustainable cultivation practices, and enhance the crop’s productivity and resilience. Furthermore, the research will contribute to strengthening the cassava value chain in Fiji, ultimately supporting food security and rural development in the region.

## Materials and methods

### Study area description

**Fig. 1:**
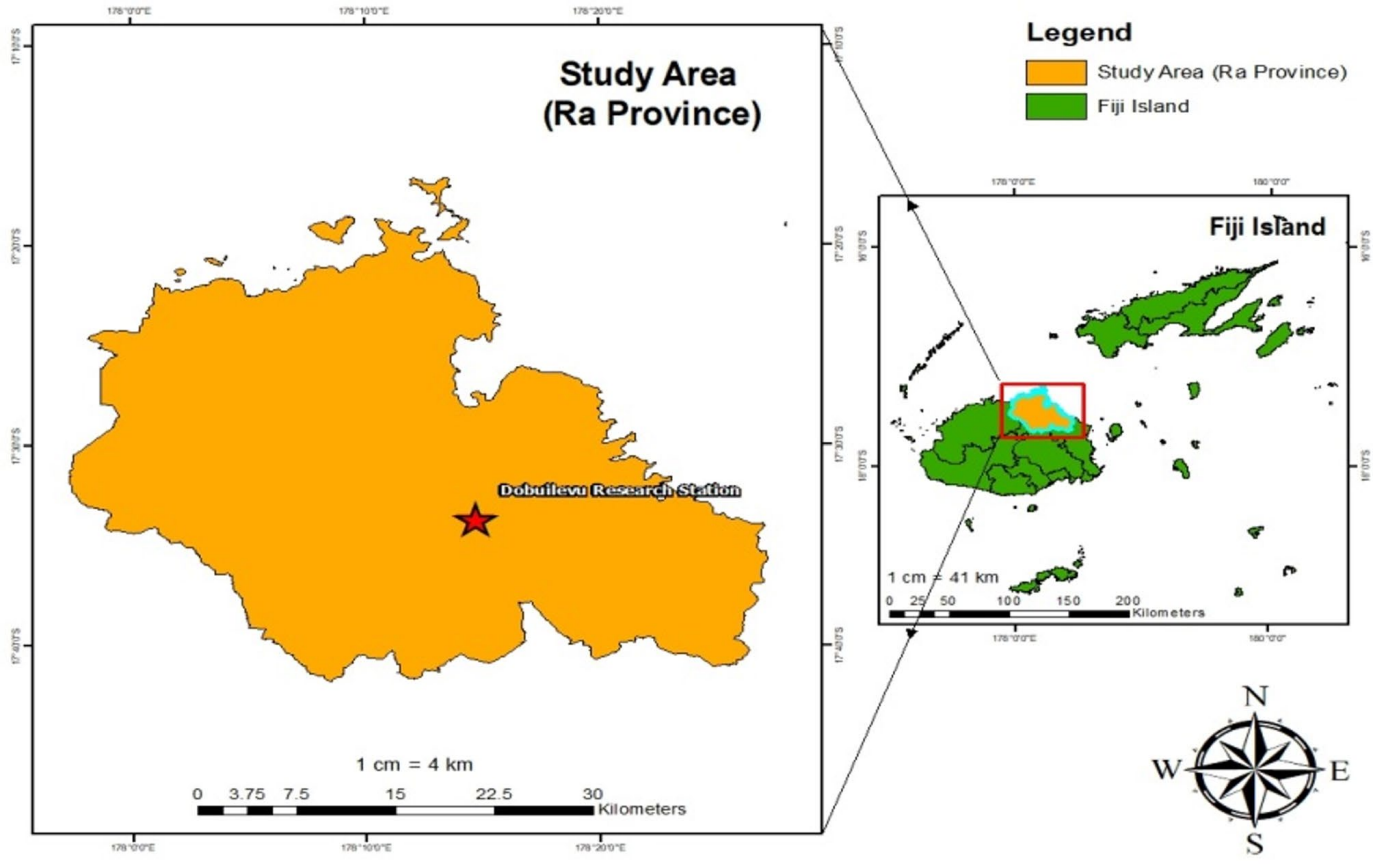
Map of the study area.

**Fig. 2:**
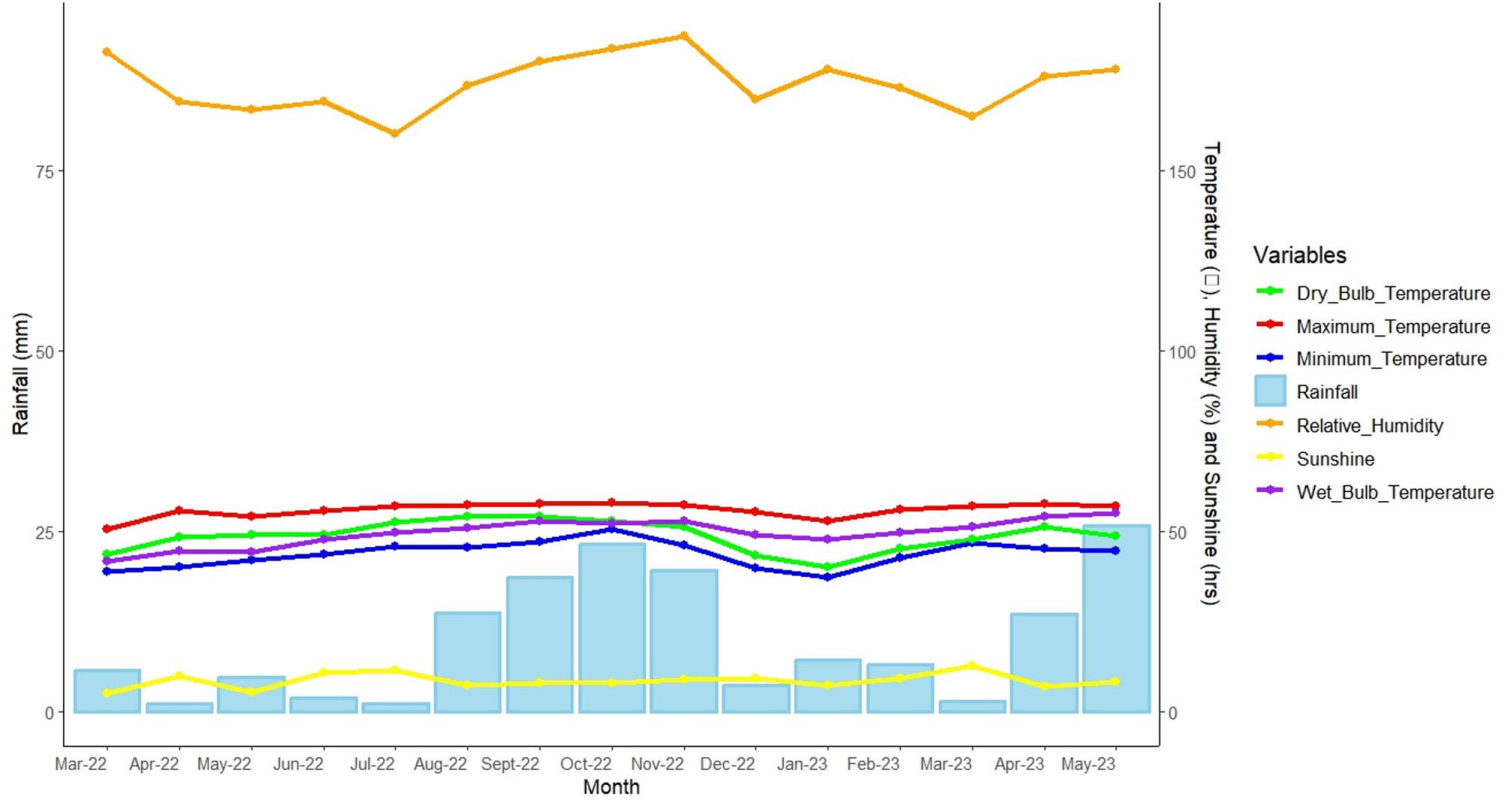
Monthly climate trends of the experimental location.

An experimental trial was carried out at the Dobuilevu Research Station in Ra Province, located northeast of Viti Levu, Fiji, during the 2022/2023 cropping season, which spanned from March to May. The research site is at an elevation of 85 m above mean sea level (m amsl), with coordinates 17°33’39” S latitude and 178°14’42” E longitude, approximately 111 km from Suva, the capital city (Fig. 1). The area experiences distinct wet and dry seasons, with the rainy season lasting from November to April and the dry season from May to October. The average annual rainfall is 2,661 mm, and temperatures range from a minimum of 20 °C to a maximum of 31 °C. Relative humidity average is 80.5% (Table 1; Fig. 2). The Dobuilevu Research Station consists of two primary physiographic features: alluvial plains and rolling to moderately steep hill terrain. The soil profile texture transitions from clay loam in the upper layer (0–50 cm) to sandy clay loam in the middle layer (50–90 cm), and loamy sand with in situ rock in the lower layer (90–120 cm) derived from basic and intermediate sedimentary rocks. The soil is slightly acidic, with very low salinity, optimal bulk density, and a warm temperature. It contains a notably high level of soil organic carbon but low available nitrogen. The available phosphorus content is moderate, while exchangeable potassium levels are low. The soil also had elevated levels of exchangeable calcium and magnesium, minimal sodium content, high exchangeable iron, exceptionally high manganese, significant copper levels, and moderate exchangeable zinc (Table 2).

**Table 1:** Meteorological data of the studied area.

Parameter	Average Value	Unit
Annual rainfall	2,661	mm
Minimum temperature	20.0	°C
Maximum temperature	31.0	°C
Relative humidity	80.5	%
Solar radiation	3.8	Hrs.

**Table 2:** Soil analysis of the studied area.

Soil properties	Value	Rating
Soil pH	5.77	Slightly acidic
Electrical conductivity (mS cm^− 1^)	0.05	Very low salinity
Bulk density (g cm^− 3^)	1.09	Optimal
Soil temperature (℃)	27.33	Warm
Soil organic carbon (%)	2.27	High
Soil available nitrogen (%)	0.08	Low
Soil available phosphorus (mg kg^− 1^)	12.33	Medium
Soil exchangeable potassium (me/100 g)	0.40	Low
Soil exchangeable calcium (me/100 g)	18.52	High
Soil exchangeable magnesium (me/100 g)	11.30	High
Soil exchangeable sodium (me/100 g)	0.04	Very low
Soil exchangeable iron (mg/kg)	30.62	High
Soil exchangeable manganese (mg/kg)	45.20	Very high
Soil exchangeable copper (mg/kg)	6.55	High
Soil exchangeable zinc (mg/kg)	1.50	Medium

### Planting material, experimental design, and layout

A total of 33 distinct cassava varieties were obtained from the cassava germplasm field at the Dobuilevu Research Station. The experimental material included stem cuttings of the 33 cassava varieties. The investigation was performed using a randomized complete block design (RCBD) with three replications, spanning a total field area of 0.47 acres (100 m x 19 m). For planting, 20 stem cuttings from each variety, each measuring 30 cm in length, were positioned in 5 m x 2 m plot sizes, with a spacing of 50 cm between plants within rows and 1 m between ridges.

### Data collection

**Fig. 3:**
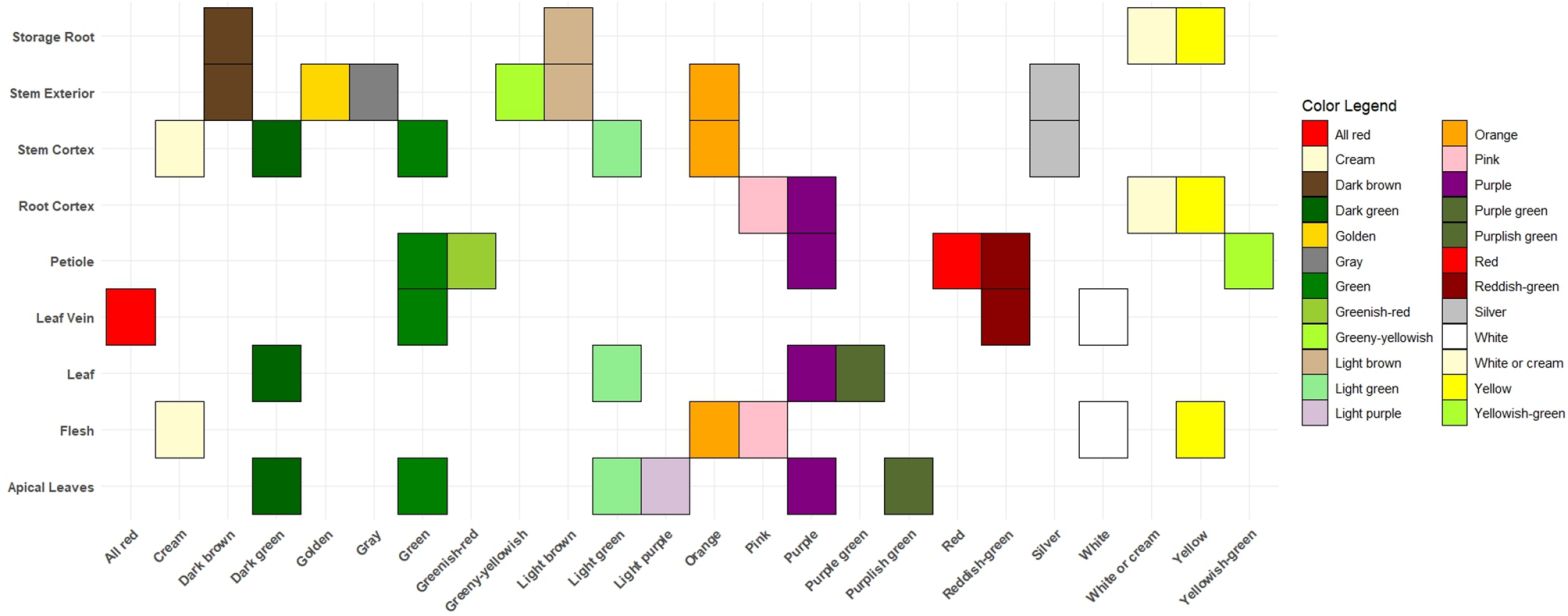
Cassava color chart representing variations in different plant parts.

The evaluation of agro-morphological traits (qualitative and quantitative), was conducted using the agro-morphological descriptors for cassava as defined by^10^. The characteristics were assessed at 3 different times, measured in months after planting (MAP). Qualitative data were collected at intervals of 3, 6, and during the harvesting stage depending on variety (7/8/9/10 MAP) (Tables 3 and 4). Figure 3 illustrates the different hues/ colors observed in various plant parts.

**Table 3:** Qualitative traits and scoring observed at 3, 6 and 7/8/9/10 MAP.

Sampling time (MAP)	Trait	Code	Scoring
3	Color of apical leaves	CAL	(1) Green; (3) Light green; (5) Dark green; (7) Purplish green; (9) Purple; (11) Light purple
6	Shape of central leaf	SCL	(1) Ovoid; (2) Elliptic-lanceolate; (3) Obovate-lanceolate; (4) Oblong-lanceolate; (5) Lanceolate; (6) Linear; (7) Pandurate; (8) Linear-piramidal; (9) Linear-pandurate; (10) Linear-hostatilobalate
6	Petiole color	PC	(1) Yellowish-green; (2) Green; (3) Reddish-green; (5) Greenish-red; (7) Red; (9) Purple
6	Leaf color	LC	(3) Light green; (5) Dark green; (7) Purple green; (9) Purple
6	Color of leaf vein	CLV	(3) Green; (5) Reddish-green in less than half of the lobe; (7) Reddish-green in more than half of the lobe; (9) All red; (11) White
7/8/9/10	Color of stem exterior	CSE	(3) Orange; (4) Greeny-yellowish; (5) Golden; (6) Light brown; (7) Silver; (8) Gray; (9) Dark brown
7/8/9/10	Branching/ Non-branching	B/N-B	(0) Non-branching; (1) Branching
7/8/9/10	Root shape	RS	(1) Conical; (2) Conical-cylindrical; (3) Cylindrical; (4) Irregular
7/8/9/10	External color of storage root	ECSR	(1) White or cream; (2) Yellow; (3) Light brown; (4) Dark brown
7/8/9/10	Color of root cortex	CRC	(1) White or cream; (2) Yellow; (3) Pink; (4) Purple
7/8/9/10	Orientation of petiole	OP	(1) Inclined upwards; (3) Horizontal; (5) Inclined downwards; (7) Irregular
7/8/9/10	Color of stem cortex	CSC	(1) Orange; (2) Light green; (3) Dark green; (4) Green; (5) Cream; (6) Silver
7/8/9/10	Color of flesh	CF	(1) White; (2) Cream; (3) Yellow; (4) Orange; (5) Pink

**Table 4:** Quantitative traits with their descriptors.

Sampling time (MAP)	Trait	Code	Techniques of measurement
6	Number of leaf lobes	NLL	Observe a leaf from the middle of the plant. Assess on five leaves and take the predominant number of lobes.
7/8/9/10	Levels of branching	LB	Number of branching points or levels.
7/8/9/10	Maturity (months)	M	Number of months to maturity.
7/8/9/10	Number of tubers	NT	Count the number of tubers per plant with average from 3 plants.
7/8/9/10	Yield (t/ha)	Y	Weigh and record the weight of the fresh tubers.
7/8/9/10	Starch content (%)	SC	AOAC method 996.11 using acid hydrolysis^17^
7/8/9/10	Iron content (mg/kg)	IC	AOAC method 999.10 using atomic absorption spectroscopy ^18^

### Data analysis

All collected data subjected to analysis were organized into an Excel spreadsheet, forming a “cassava agro-morphology” matrix. To evaluate the genetic variation of agro-morphological traits among the cassava varieties, multivariate analysis was performed. Separate analyses were conducted on the 33 × 13 qualitative and the 33 × 7 quantitative traits using principal component analysis (PCA). Eigenvalues and load coefficients were extracted during the PCA. The contribution of each trait to the variance explained by the principal components (PCs) was assessed using an eigenvector cutoff value of 0.30, as recommended by^19^. The PCA and correlation matrices were utilized to explore trait relationships. Descriptive statistics for both types of data were calculated using R software version 3.6.1^20^. An ascending and circular hierarchical clustering approach was used to visualize the structure and organization of morphological variability through a dendrogram. A scree plot identified the optimal number of PCs, highlighting each component’s contribution to the overall variance in the agro-morphological data.

## Results

### Evaluating cassava varieties based on qualitative traits: correlation, PCA, and descriptive statistics

**Fig. 4:**
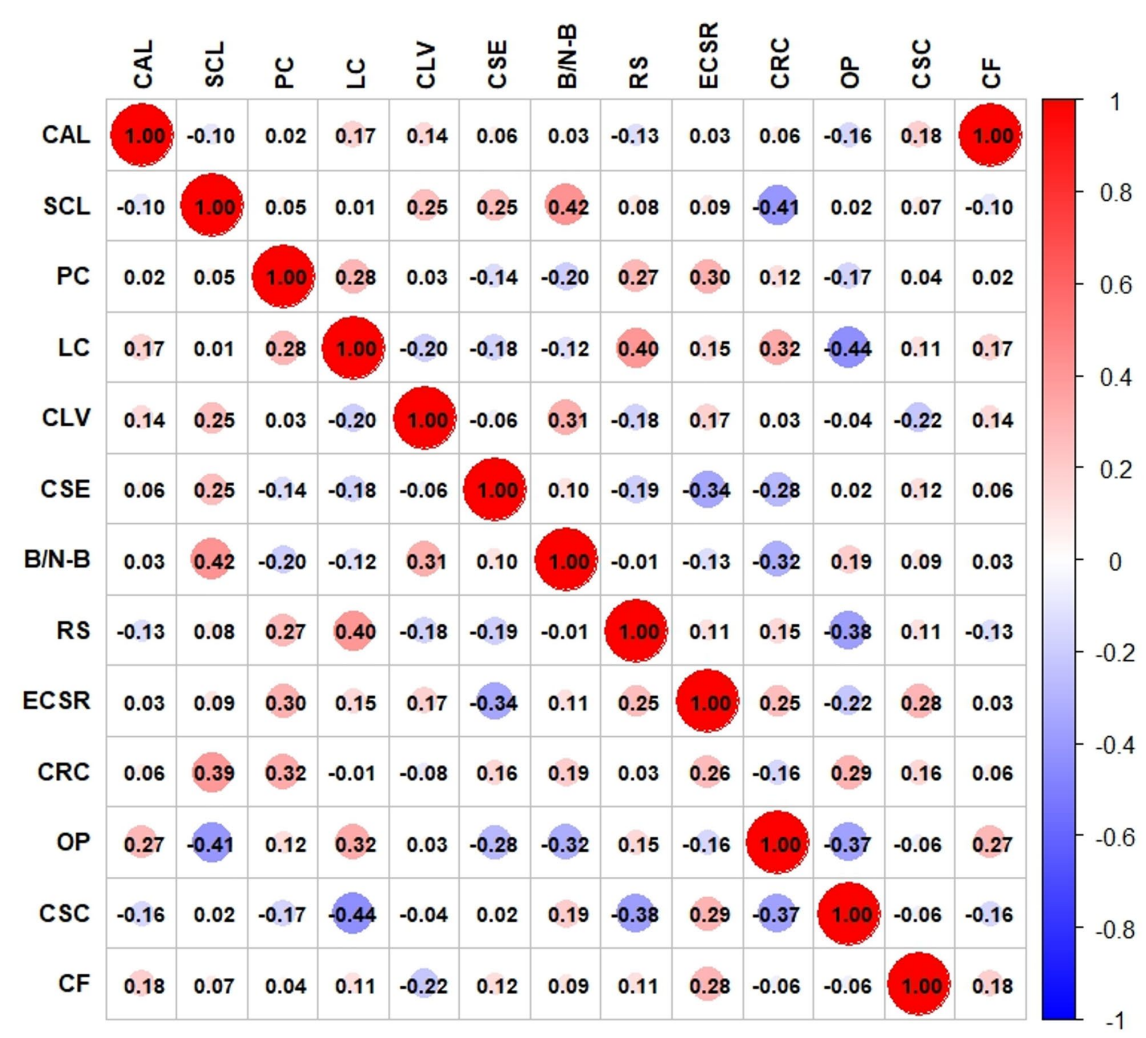
Correlation coefficient among qualitative traits of 33 cassava varieties.

The correlation analysis of 13 qualitative traits revealed several positive relationships among characteristics. Apical leaf color was correlated with leaf color *(r = 0.17)*. Central leaf shape strongly correlation with branching behavior *(r = 0.42)*. Petiole color was positively associated with root cortex color *(r = 0.32)*, and leaf color was correlated with root shape *(r = 0.40)*. Leaf vein color was linked to branching *(r = 0.31)*, while stem exterior color had a positive relationship with root cortex color *(r = 0.16)*. Branching behavior was associated with stem cortex color *(r = 0.19)*, and root shape correlated with petiole orientation *(r = 0.15)*. External storage root color showed a positive correlation with root cortex color *(r = 0.26)*, and root cortex color was linked to stem cortex color *(r = 0.29)*. Apical leaf color correlated with petiole orientation *(r = 0.27)*, and central leaf shape showed a strong relationship with root cortex color *(r = 0.39)*. Petiole color correlated with external storage root color *(r = 0.30)*, while leaf color had a positive association with petiole orientation *(r = 0.32)*. Leaf vein color correlated with external storage root color *(r = 0.17)*, and stem cortex color was linked to flesh color *(r = 0.12)*. Branching behavior had a positive relationship with root cortex color *(r = 0.19)*, and root shape correlated with flesh color *(r = 0.11)*. External root color was associated with flesh color *(r = 0.28)*, and root cortex color correlated with flesh color *(r = 0.16)*. Apical leaf color showed a positive relationship with flesh color *(r = 0.18)*. Central leaf shape correlated with leaf vein color *(r = 0.25)*, and petiole color had a positive relationship with leaf color *(r = 0.28)*. Leaf color correlated with external storage root color *(r = 0.15)*, and stem exterior color was associated with branching *(r = 0.10)*. Root shape correlated with external root color *(r = 0.11)*, while external root color showed a positive relationship with petiole orientation *(r = 0.25)*. Apical leaf color was linked to leaf vein color *(r = 0.14)*, and central leaf shape correlated with stem exterior color *(r = 0.25)*. Petiole color had a positive association with root shape *(r = 0.27)*, leaf color correlated with flesh color *(r = 0.11)*, and petiole color showed a weak correlation with petiole orientation *(r = 0.12)* (Fig. 4). On the other hand, negative correlations were observed between several traits. These include: apical leaf color correlating with central leaf shape *(r = − 0.10)*, apical leaf color correlating with root shape *(r = − 0.13)*, and apical leaf color correlating with stem cortex color *(r = − 0.16)*. Central leaf shape showed a negative correlation with petiole orientation *(r = − 0.41)*. Petiole color was negatively correlated with stem exterior color *(r = − 0.14)*, branching behavior *(r = − 0.20)*, and stem cortex color *(r = − 0.17)*. Leaf color was negatively associated with leaf vein color *(r = − 0.20)*, stem exterior color *(r = − 0.18)*, branching behavior *(r = − 0.12)*, and stem cortex color *(r = − 0.44)*. Leaf vein color had negative correlations with root shape *(r = − 0.18)* and flesh color *(r = − 0.22)*. Stem exterior color showed negative relationships with root shape *(r = − 0.19)*, external root color *(r = − 0.34)*, and petiole orientation *(r = − 0.28)*. Branching behavior was negatively correlated with external root color *(r = − 0.13)* and petiole orientation *(r = − 0.32)*. Root shape had a negative relationship with stem cortex color *(r = − 0.38)*, and external root color was negatively correlated with stem cortex color *(r = − 0.22)*. Petiole orientation was negatively associated with stem cortex color *(r = − 0.37)*.

**Fig. 5:**
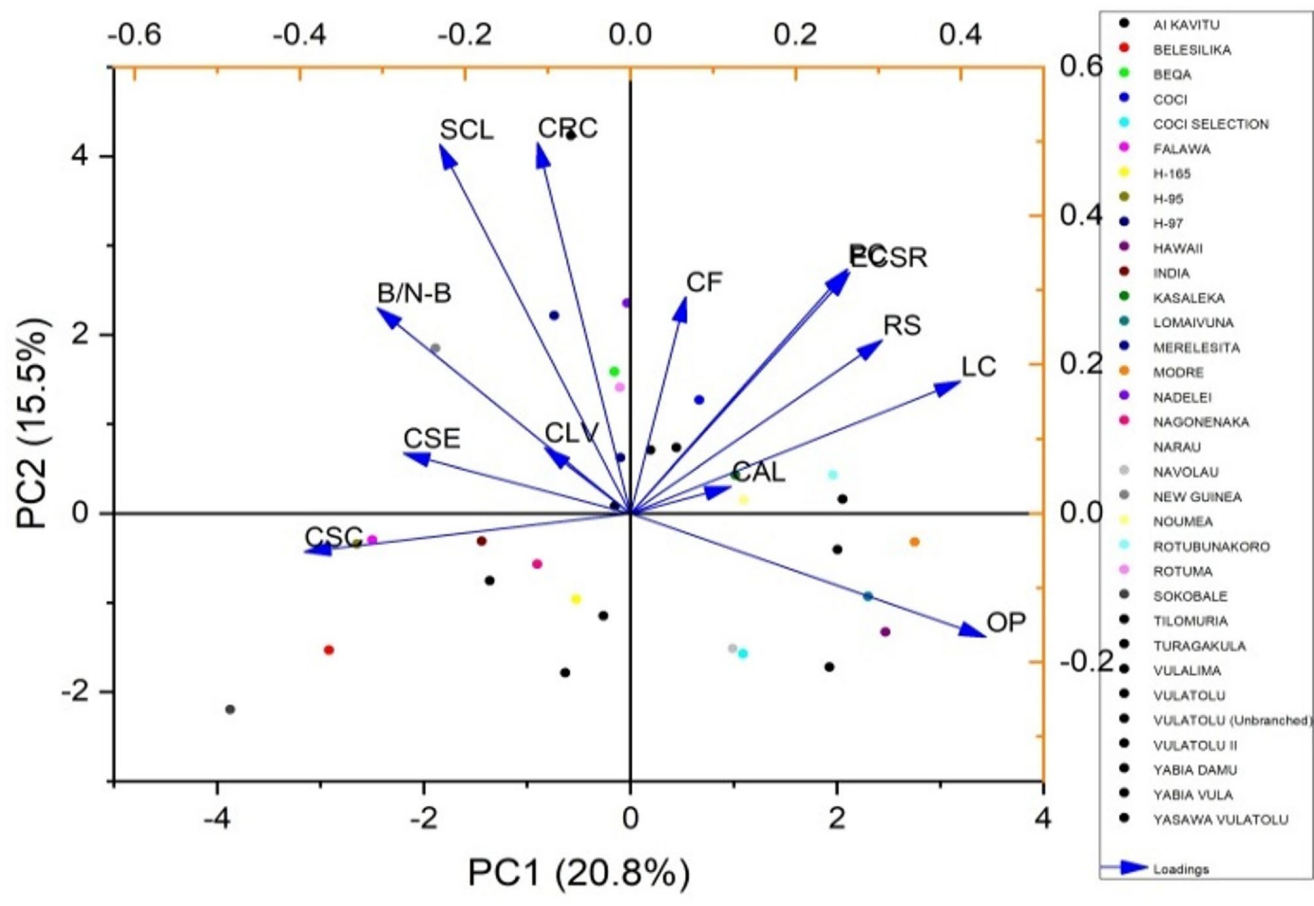
PCA biplot showing contributions of qualitative traits to the variability among cassava varieties.

**Fig. 6:**
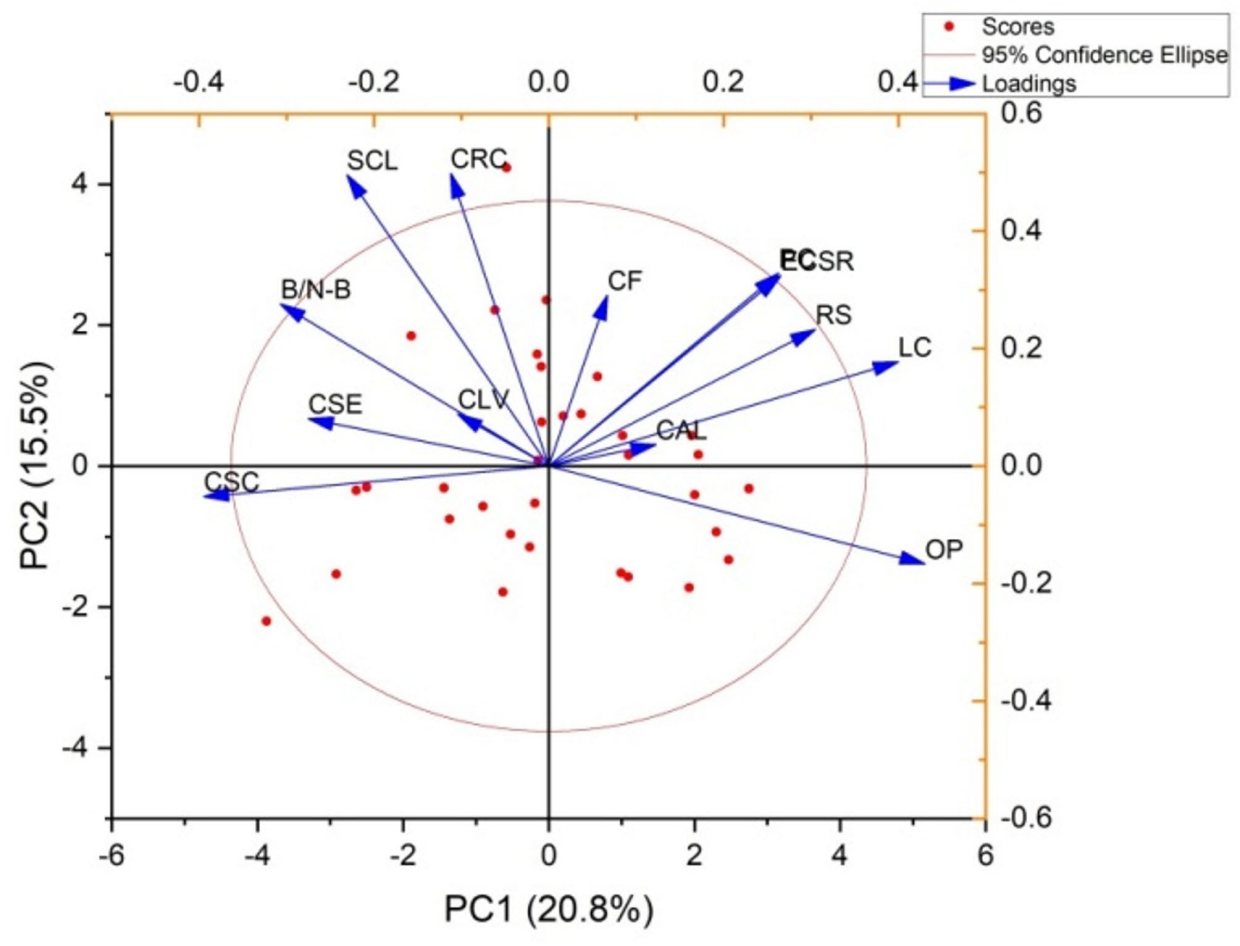
PCA biplot illustrating the distribution of cassava varieties based on contributions of qualitative traits.

The eigenvalues, percentage of variation, and PCs for the various traits are outlined in Table 5. Two PCs were identified, collectively accounting for 36.31% of the overall variation among the cassava varieties (Fig. 5). The color of apical leaves had the highest eigenvalue of 2.706, explaining 20.82% of the variation, with a cumulative variance of 20.82%. The shape of the central leaf contributed an eigenvalue of 2.014, which accounted for 15.49% of the total variation, raising the cumulative variance to 36.31%. The petiole color trait showed an eigenvalue of 1.456, explaining 11.20% of the variance, leading to a cumulative variance of 47.51%. Leaf color contributed 10.20% to the total variation, with an eigenvalue of 1.327, resulting in a cumulative variance of 57.71%. The color of leaf veins had an eigenvalue of 1.286, accounting for 9.90% of the variance, with a cumulative variance of 67.61%. The color of stem exterior explained 7.51% of the variation, with an eigenvalue of 0.976, which increased the cumulative variance to 75.11%. The branching/non-branching trait contributed an eigenvalue of 0.841, explaining 6.47% of the variance, bringing the cumulative variance to 81.59%. Root shape showed an eigenvalue of 0.597, accounting for 4.59% of the total variance, resulting in a cumulative variance of 86.18%. The external color of storage root explained 4.29% of the variation, with an eigenvalue of 0.558, bringing the cumulative variance to 90.47%. The color of root cortex had an eigenvalue of 0.427, accounting for 3.28% of the variance, with a cumulative variance of 93.75%. The orientation of petiole contributed 2.66% of the variance, with an eigenvalue of 0.346, increasing the cumulative variance to 96.41%. The color of stem cortex contributed an eigenvalue of 0.274, explaining 2.11% of the total variation, raising the cumulative variance to 98.52%. The color of flesh accounted for the least amount of variation, with an eigenvalue of 0.192, explaining 1.48% of the variance, resulting in a total cumulative variance of 100% (Fig. 6).

**Table 5:** Principal component analysis for the qualitative traits of the cassava varieties.

Trait	PC1	PC2	Eigenvalue	Percentage of variance (%)	Cumulative variance (%)
CAL	0.122	0.036	2.706	20.82	20.82
SCL	-0.231	0.496	2.014	15.49	36.31
PC	0.263	0.328	1.456	11.20	47.51
LC	0.400	0.178	1.327	10.20	57.71
CLV	-0.104	0.088	1.286	9.90	67.61
CSE	-0.275	0.081	0.976	7.51	75.11
B/N-B	-0.307	0.276	0.841	6.47	81.59
RS	0.305	0.233	0.597	4.59	86.18
ECSR	0.265	0.324	0.558	4.29	90.47
CRC	-0.113	0.498	0.427	3.28	93.75
OP	0.430	-0.166	0.346	2.66	96.41
CSC	-0.395	-0.051	0.274	2.11	98.52
CF	0.067	0.291	0.192	1.48	100.00

CAL, color of apical leaves; SCL, shape of central leaf; PC, petiole color; LC, leaf color; CLV, color of leaf vein; CSE, color of stem exterior; B/N-B, branching/ non-branching; RS, root shape; ECSR, external color of storage root; CRC, color of root cortex; OP, orientation of petiole; CSC, color of stem cortex; CF, color of flesh.

**Fig. 7:**
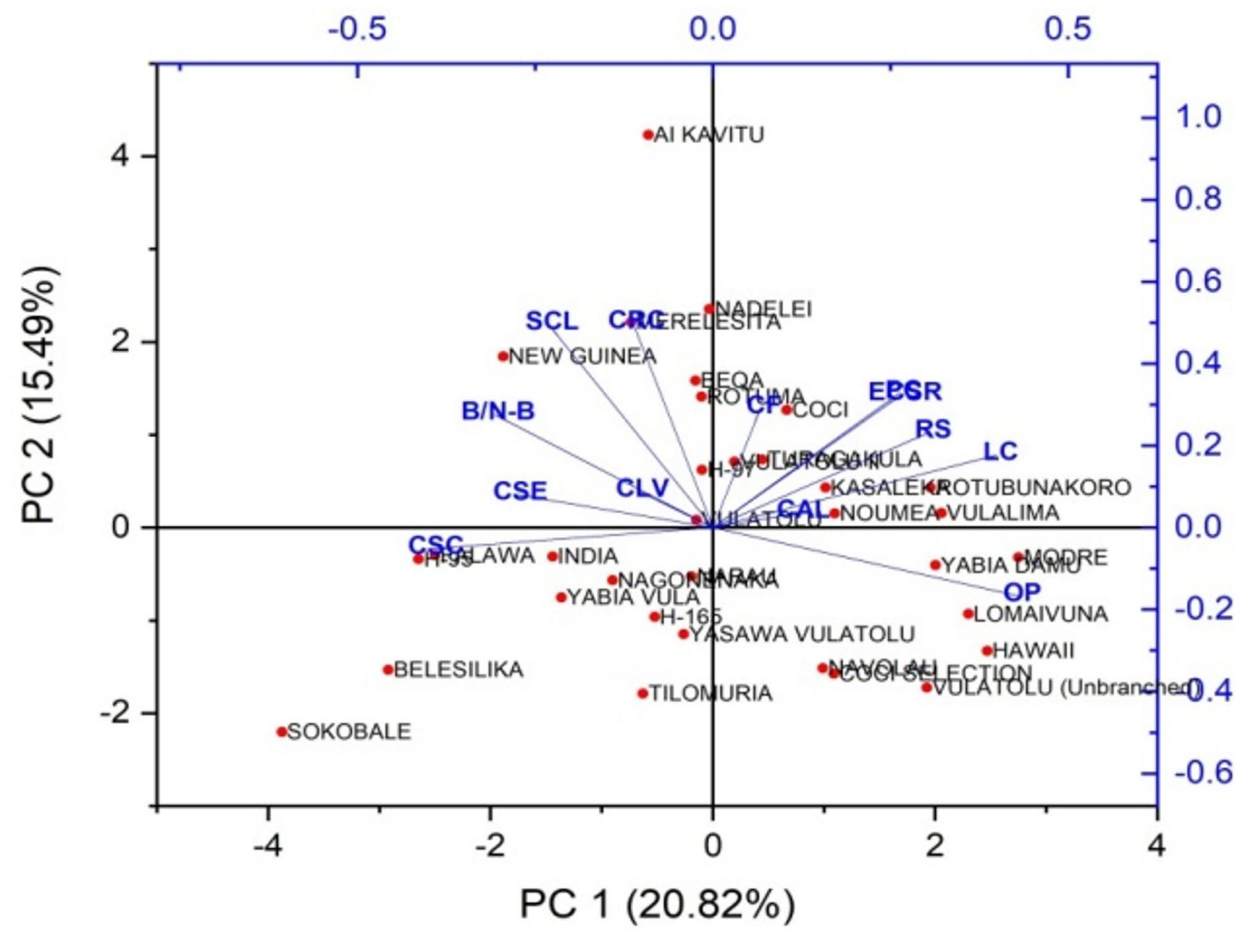
PCA biplot highlighting the relationships between cassava varieties and qualitative traits, focusing on the clustering and contributions of variables along the principal components.

**Fig. 8:**
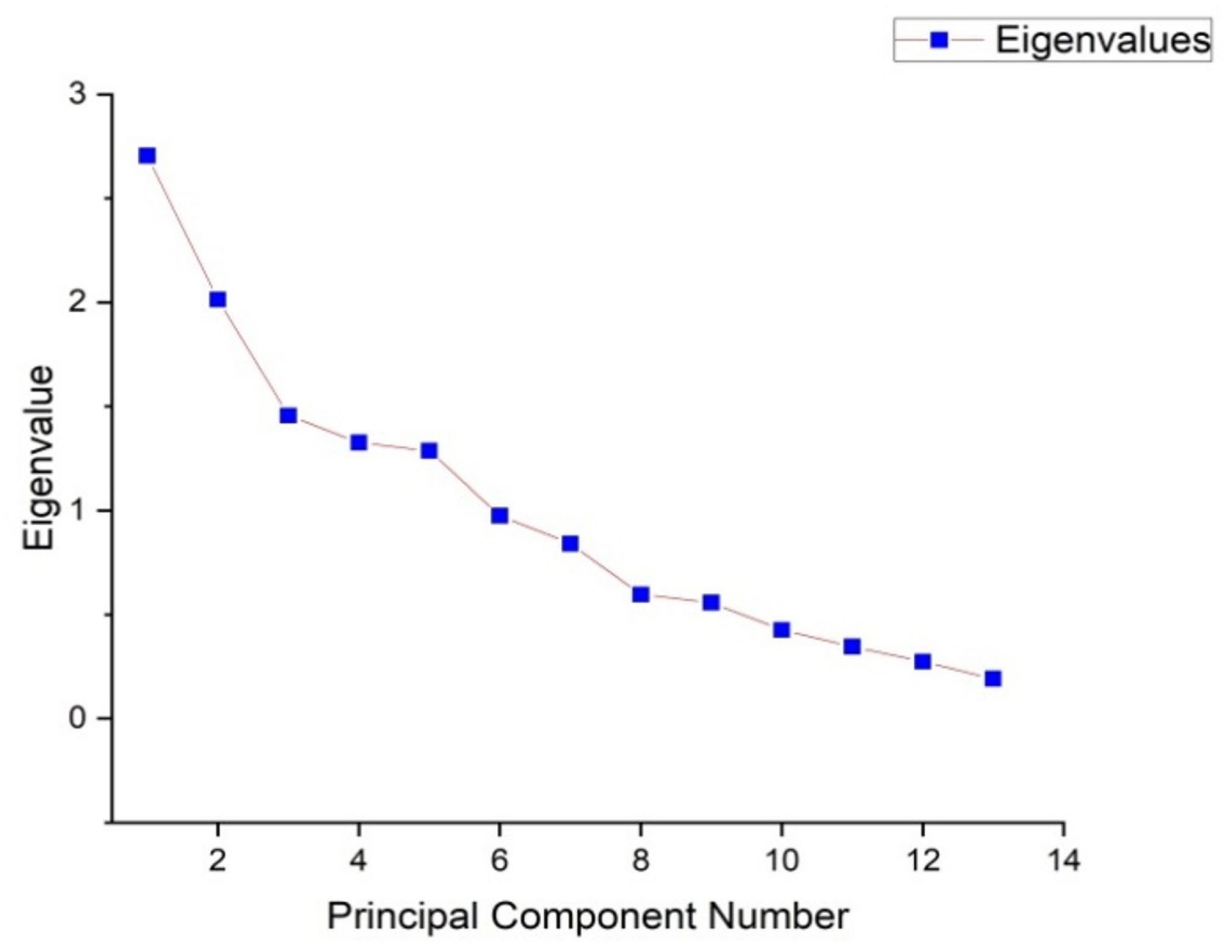
Scree plot displaying eigenvalues against the principal component numbers, demonstrating the variance explained by each component and indicating the importance of the first few components.

Figures 7 and 8 illustrate PCA biplots for 33 cassava varieties based on the contributions of 13 qualitative traits. The biplots effectively capture the variation in the data along PC1 and PC2, emphasizing the relationships between cassava varieties and qualitative traits. They highlight the contributions of each variable and illustrate how the traits cluster along the PCs. The PCs illustrates the contributions of each trait. In PC1, the trait orientation of the petiole had the highest positive loading of 0.430. Leaf color also contributed significantly to PC1 with a loading of 0.400 along the axis. Other traits such as petiole color (0.263) and color of stem exterior (-0.275) also had moderate loadings on PC1. Traits like branching/ non-branching (-0.307) and color of stem cortex (-0.395) showed negative loadings on PC1. In PC2, the shape of central leaf and color of root cortex exhibited the highest positive loadings, with values of 0.496 and 0.498, respectively. Root shape (0.233) and external color of storage root (0.324) also had positive loadings on PC2. Traits such as color of leaf vein (0.088) and color of flesh (0.291) had relatively smaller and more mixed contributions to PC2. The PC1 had significant positive associations with orientation of petiole, leaf color and petiole color. Traits such as branching/ non-branching and color of stem cortex had negative loadings on PC1. The PC2 component was positively correlated with the shape of central leaf, color of root cortex, and external color of storage root. However, the associations between PC2 and the color of stem exterior and leaf color were comparatively weaker.

**Fig. 9:**
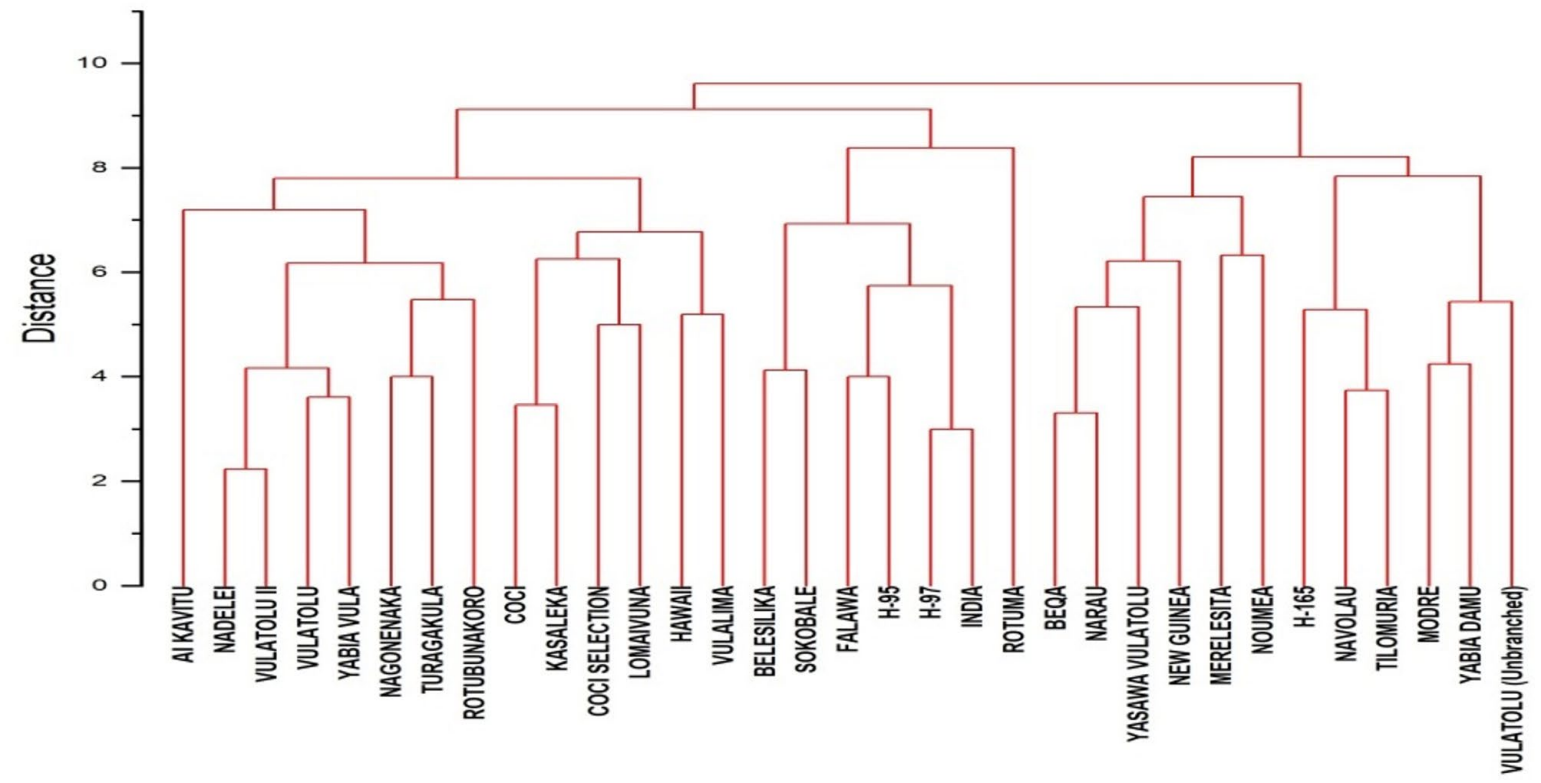
Dendrogram showing relationships among 33 varieties of cassava using 13 qualitative agro-morphological traits.

The 33 cassava varieties were classified hierarchically based on their qualitative traits. The varieties were grouped into three categories with similar characteristics. The genetic similarity for the 13 qualitative traits ranged from 0 to 10. The average similarity score was 5. The cassava varieties formed three distinct clusters. Cluster I included 14 varieties. Cluster III contained 12 varieties. Cluster II had 7 varieties (Fig. 9).

The variation among the 33 cassava varieties for the qualitative traits is summarized in Table 6. The color of apical leaves had an average value of 5.48, with a standard deviation of 3.36, ranging from 1.00 to 11.00, and a median of 5.00. The shape of the central leaf had a mean of 3.91, with a standard deviation of 1.31, ranging from 2.00 to 6.00, and a median of 5.00. The petiole color averaged 4.27, with a standard deviation of 2.34, values spanning from 1.00 to 9.00, and a median of 4.00. The leaf color had a mean of 4.52 and a low standard deviation of 0.87, indicating uniformity, with a median of 5.00 and values between 3.00 and 5.00. The color of the leaf vein showed the highest mean of 7.67, with a standard deviation of 3.07, ranging from 3.00 to 11.00, and a median of 9.00. The color of the stem exterior had an average of 5.67, with a standard deviation of 1.91, values ranging from 3.00 to 9.00, and a median of 7.00. For branching/non-branching, the mean was 0.79, with a low standard deviation of 0.42, indicating values predominantly near 0 or 1. The root shape averaged 2.70, with a standard deviation of 0.64, a consistent median of 3.00, and values ranging from 1.00 to 3.00. The external color of the storage root had a mean of 3.09, with values from 1.00 to 4.00 and a median of 4.00. The color of the root cortex had a mean of 1.24, with a standard deviation of 0.66, values ranging from 1.00 to 3.00. The orientation of petiole had an average of 2.76, with a standard deviation of 1.79, values between 1.00 and 7.00, and a median of 3.00. The color of the stem cortex had a mean of 2.67, with a standard deviation of 1.08, and most values were close to 2.00. Lastly, the color of flesh had a mean of 1.24, similar to the color of root cortex, with values ranging from 1.00 to 3.00 and a low standard deviation of 0.61.

**Table 6:** Descriptive statistics of qualitative traits for the cassava varieties.

Trait	Mean	Standard Deviation	Sum	Minimum	Median	Maximum
CAL	5.48	3.36	181.00	1.00	5.00	11.00
SCL	3.91	1.31	129.00	2.00	5.00	6.00
PC	4.27	2.34	141.00	1.00	4.00	9.00
LC	4.52	0.87	149.00	3.00	5.00	5.00
CLV	7.67	3.07	253.00	3.00	9.00	11.00
CSE	5.67	1.91	187.00	3.00	7.00	9.00
B/N-B	0.79	0.42	26.00	0.00	1.00	1.00
RS	2.70	0.64	89.00	1.00	3.00	3.00
ECSR	3.09	1.33	102.00	1.00	4.00	4.00
CRC	1.24	0.66	41.00	1.00	1.00	3.00
OP	2.76	1.79	91.00	1.00	3.00	7.00
CSC	2.67	1.08	88.00	2.00	2.00	6.00
CF	1.24	0.61	41.00	1.00	1.00	3.00

CAL, color of apical leaves; SCL, shape of central leaf; PC, petiole color; LC, leaf color; CLV, color of leaf vein; CSE, color of stem exterior; B/N-B, branching/ non-branching; RS, root shape; ECSR, external color of storage root; CRC, color of root cortex; OP, orientation of petiole; CSC, color of stem cortex; CF, color of flesh.

Table 7 revealed diverse phenotypic expressions with the 33 cassava varieties. The color of apical leaves showed 6 variations, with green, dark green, and purple being the most frequent (around 24–27%), and light purple the least (3.03%). The shape of the central leaf presented five variations, lanceolate being the most common (48.48%) and linear the least (3.03%). Petiole color also displayed five variations, with red being the most frequent (30.3%), followed by reddish-green and greenish-red (both 24.24%), and while purple was least frequent (3.03%). Leaf color had two variations, with dark green dominating (75.76%). The color of the leaf vein showed five variations, white being the most frequent (33.33%). The color of the stem exterior had four variations, with silver being the most common (57.58%). Branching/ Non-branching was predominantly branching (78.79%). Root shape was mostly cylindrical (78.79%). The external color of the storage root was predominantly dark brown (63.64%). The color of the root cortex was most often cream-colored (72.73%). Orientation of the petiole was either horizontal or inclined upwards in equal frequency (39.39%). The color of the stem cortex was primarily silver (63.64%). Finally, the color of the flesh was predominantly white (84.85%).

**Table 7:** Trait frequencies and percentage distribution for the cassava varieties.

Trait	Categories	Frequencies	Percent distribution (%)
CAL	Green, Dark green, Purple, Purplish green, Light green, Light purple	9, 8, 8, 5, 1, 2	27.27, 24.24, 24.24, 15.15, 6.06, 3.03
SCL	Lanceolate, Elliptic-lanceolate, Obovate-lanceolate, Oblong-lanceolate, Linear	16, 7, 7, 2, 1	48.48, 21.21, 21.21, 6.06, 3.03
PCR	Red, Reddish-green, Greenish-red, Yellowish-green, Purple	10, 8, 8, 6, 1	30.3, 24.24, 24.24, 18.18, 3.03
LC	Dark green, Light green	25, 8	75.76, 24.24
CLV	White, Green, Red, Reddish-green in more than half of the lobe, Reddish-green in less than half of the lobe	11, 6, 6, 5, 5	33.33, 18.18, 18.18, 15.15, 15.15
CSE	Silver, Orange, Golden, Dark brown	19, 10, 3, 1	57.58, 30.3, 9.09, 3.03
B/N-B	Branching, Non-branching	26, 7	78.79, 21.21
RS	Cylindrical, Colonial-cylindrical, Conical, Conical-cylindrical	26, 3, 3, 1	78.79, 9.09, 9.09, 3.03
ECS	Dark brown, Cream, Light brown	21, 9, 3	63.64, 27.27, 9.09
CRC	Cream, Pink, White	24, 5, 4	72.73, 15.15, 12.12
OP	Horizontal, Inclined upwards, Inclined downwards, Irregular	13, 13, 5, 2	39.39, 39.39, 15.15, 6.06
CSC	Silver, Light green, Green, Dark green, Cream	21, 6, 3, 2, 1	63.64, 18.18, 9.09, 6.06, 3.03
CF	White, Yellow, Cream	28, 3, 2	84.85, 9.09, 6.06

CAL, color of apical leaves; SCL, shape of central leaf; PC, petiole color; LC, leaf color; CLV, color of leaf vein; CSE, color of stem exterior; B/N-B, branching/ non-branching; RS, root shape; ECSR, external color of storage root; CRC, color of root cortex; OP, orientation of petiole; CSC, color of stem cortex; CF, color of flesh.

### Evaluating cassava varieties based on quantitative traits: descriptive statistics, correlation and PCA

Descriptive statistics for the quantitative traits for the 33 cassava varieties are presented in Table 8. The trait number of leaf lobes had an average value of 6.82, with a standard deviation of 1.05. The values ranged from 3.00 to 9.00, and the median was 7.00. The levels of branching had a mean of 2.09 and a standard deviation of 1.57, with values ranging from 0.00 to 4.00 and a median of 2.00. For maturity, the mean was 9.32, with a low standard deviation of 0.53, indicating consistent values around 9.50, ranging from 7.50 to 9.50. The number of tubers had an average of 9.46 and a standard deviation of 1.25, with a range from 8.00 to 12.00 and a median of 9.00. The yield trait had a mean of 25.40, with a standard deviation of 1.87. The values varied from 23.00 to 30.00, with a median of 25.00. The starch content had an average of 22.94, with a standard deviation of 5.71. The range was from 11.73 to 32.70, with a median of 23.65. For iron content, the mean was 24.93, with a standard deviation of 11.72, and values ranged from 6.35 to 48.07, with a median of 22.96.

**Table 8:** Descriptive statistics of 7 quantitative characteristics of 33 cassava varieties.

Trait	Mean	Standard Deviation	Sum	Minimum	Median	Maximum
NLL	6.82	1.05	225.00	3.00	7.00	9.00
LB	2.09	1.57	69.00	0.00	2.00	4.00
M	9.32	0.53	307.50	7.50	9.50	9.50
NT	9.46	1.25	312.00	8.00	9.00	12.00
Y	25.40	1.87	838.00	23.00	25.00	30.00
SC	22.94	5.71	757.09	11.73	23.65	32.70
IC	24.93	11.72	797.80	6.35	22.96	48.07

NLL, number of leaf lobes; LB, levels of branching; M, maturity (months); NT, number of tubers; Y, yield (t/ha); SC, starch content (%); IC, iron content (mg/kg).

**Fig. 10:**
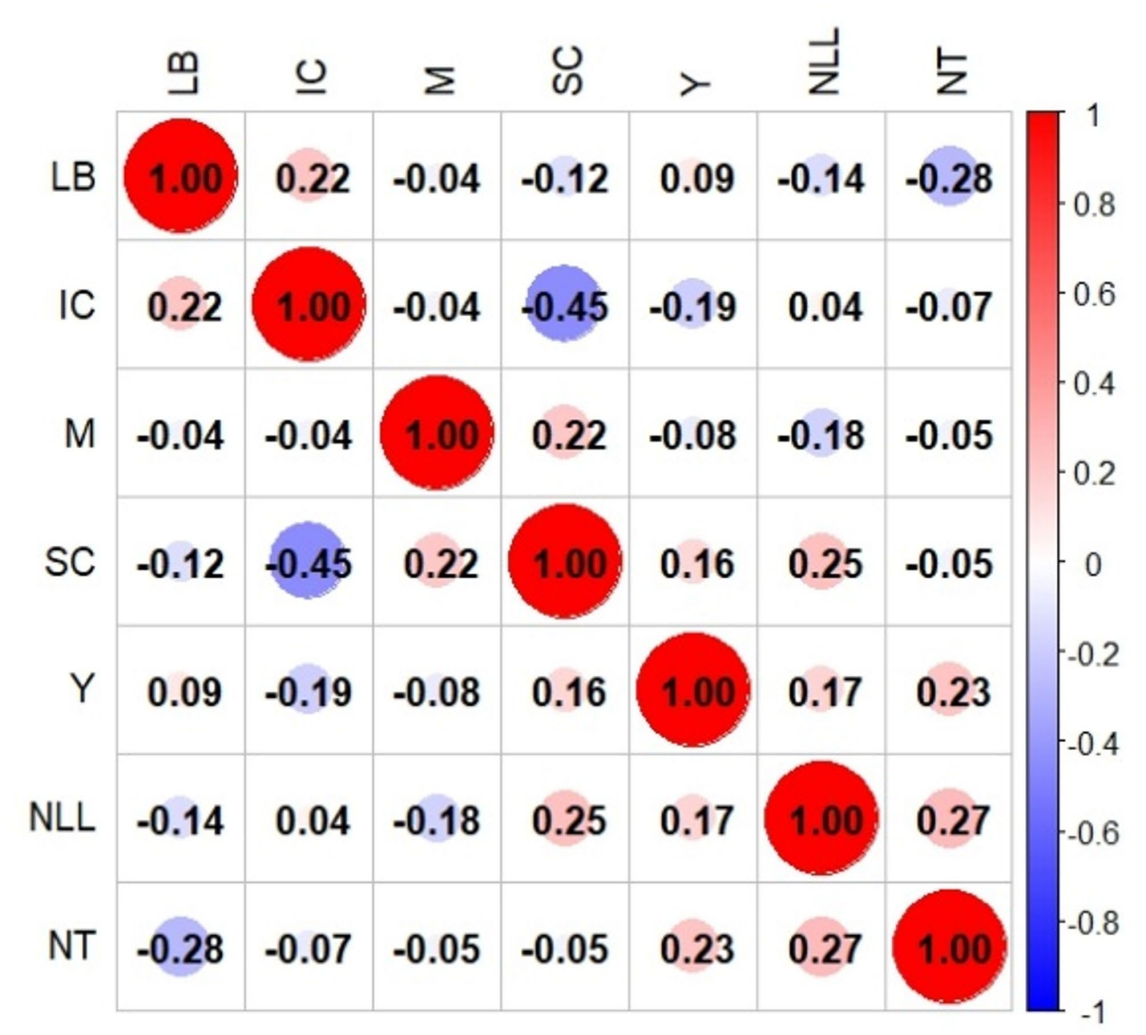
Correlation matrix heatmap among 7 quantitative traits of 33 cassava varieties.

Correlation analysis of 7 quantitative traits indicated several positive relationships. The number of leaf lobes showed positive correlations with the number of tubers *(r = 0.27)*, yield *(r = 0.17)*, and starch content *(r = 0.25)*. There was also a positive correlation between levels of branching and iron content *(r = 0.22)*, maturity and starch content *(r = 0.22)*, number of tubers and yield *(r = 0.23)*, and yield and starch content *(r = 0.16)*. On the other hand, negative correlations were found between the number of leaf lobes and levels of branching *(r = − 0.14)*, number of leaf lobes and maturity *(r = − 0.18)*, levels of branching and number of tubers *(r = − 0.28)*, levels of branching and starch content *(r = − 0.12)*, maturity and yield *(r = − 0.08)*, number of tubers and iron content *(r = − 0.07)*, yield and iron content *(r = − 0.19)*, and starch content and iron content *(r = − 0.45)* (Fig. 10).

**Fig. 11:**
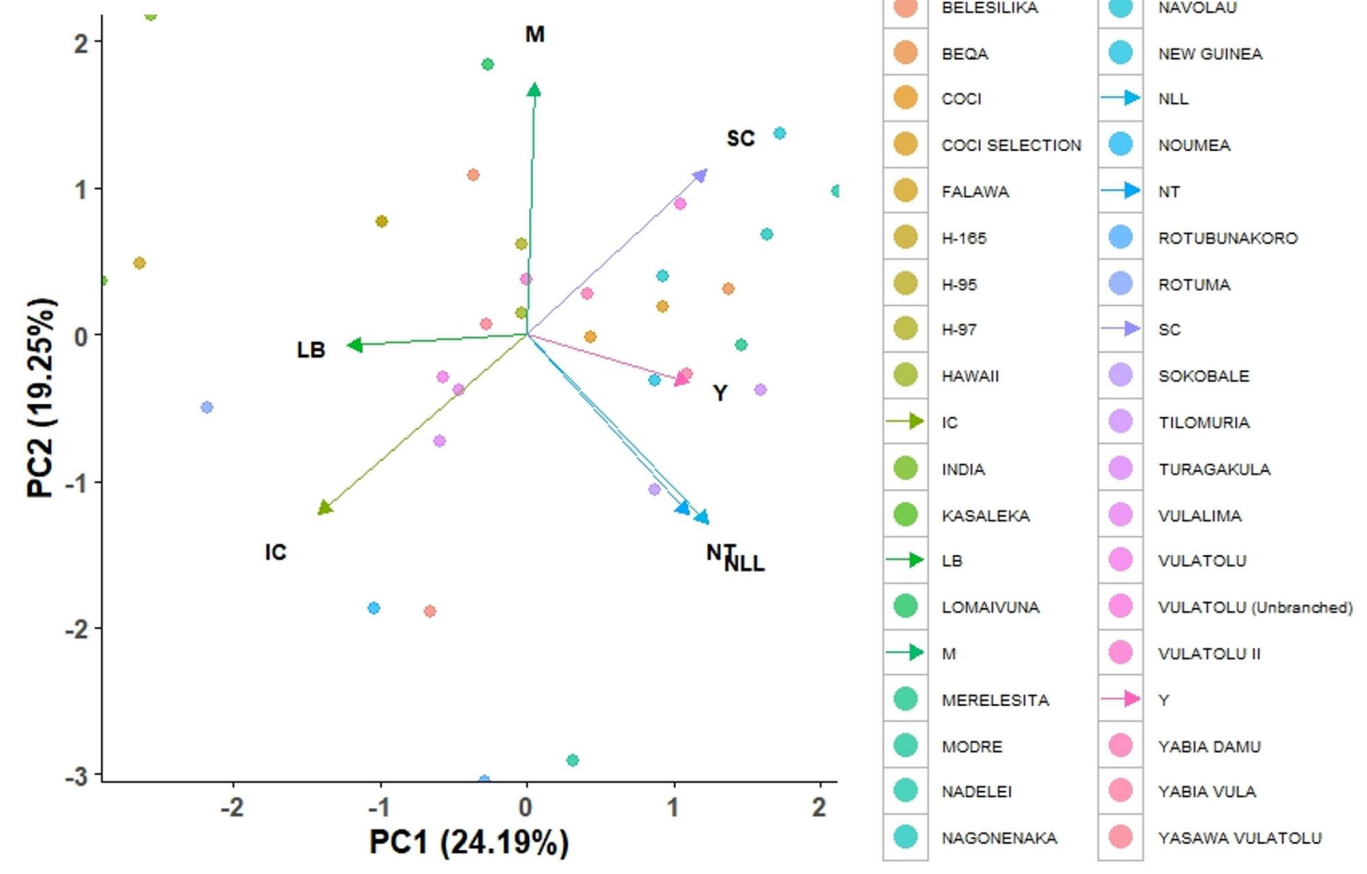
PCA biplot showing contributions of 7 quantitative traits to the variability among 33 cassava varieties.

The eigenvalues, percentage variations, and PCs for the various quantitative traits are presented in Table 9. The 2 PCs identified accounted for 43.45% of the overall variation in the traits (Fig. 11). The number of leaf lobes had the highest eigenvalue of 1.693, explaining 24.19% of the total variance, with a cumulative variance of 24.19%. The levels of branching trait had an eigenvalue of 1.348, contributing 19.25% to the variance, resulting in a cumulative variance of 43.45%. The maturity trait exhibited an eigenvalue of 1.047, accounting for 14.96% of the variance, bringing the cumulative variance to 58.41%. The number of tubers had an eigenvalue of 1.007, explaining 14.38% of the variance, leading to a cumulative variance of 72.79%. The yield trait showed an eigenvalue of 0.855, explaining 12.22% of the variance, with the cumulative variance reaching 85.01%. The starch content had an eigenvalue of 0.658, explaining 9.39% of the variance. This brought the cumulative variance to 94.40%. The iron content contributed the least, with an eigenvalue of 0.392, explaining 5.60% of the variance. This resulted in a cumulative variance of 100%.

**Table 9:** Principal component analysis for the quantitative traits of the cassava varieties.

Trait	PC1	PC2	Eigenvalue	Percentage of variance (%)	Cumulative variance (%)
NLL	1.693	24.191	1.693	24.19	24.19
LB	1.348	19.254	1.348	19.25	43.45
M	1.047	14.961	1.047	14.96	58.41
NT	1.007	14.381	1.007	14.38	72.79
Y	0.855	12.219	0.855	12.22	85.01
SC	0.658	9.3938	0.658	9.39	94.40
IC	0.392	5.601	0.392	5.60	100.00

NLL, number of leaf lobes; LB, levels of branching; M, maturity (months); NT, number of tubers; Y, yield (t/ha); SC, starch content (%); IC, iron content (mg/kg).

**Fig. 12:**
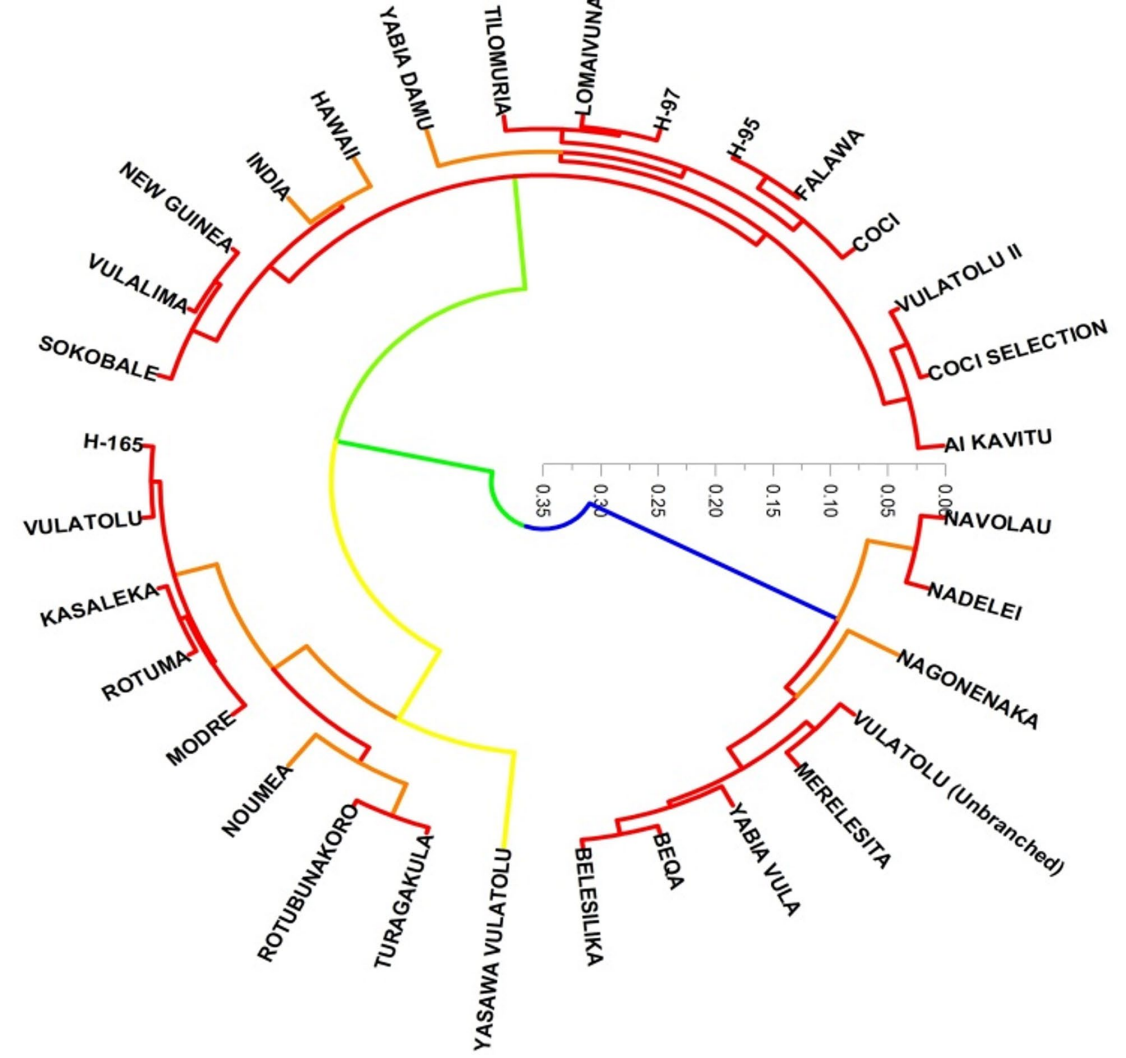
Genetic relationships among the 33 cassava varieties.

The dendrogram analysis reveals approximately 5 distinct clusters of varieties. A large and diverse group (Cluster 1) includes COCI, KASALEKA, COCI SELECTION, VULALIMA, HAWAII, LOMAIVUNA, ROTUBUNAKORO, MODRE, and YABIA DAMU. Cluster 2 comprises VULATOLU (Unbranched), H-165, NAVOLAU, TILOMURIA, BEQA, and NARAU. A tightly knit cluster (Cluster 3) is formed by FALAWA, H-95, and H-97. A more distinct cluster (Cluster 4) contains NEW GUINEA, MERELESITA, NOUMEA, ROTUMA, and INDIA. Finally, a smaller, separate cluster (Cluster 5) groups various “VULATOLU” variations: VULATOLU, TURAGAKULA, YABIA VULA, VULATOLU II, NADELEI, NAGONENAKA, AI KAVITU, and YASAWA VULATOLU (Fig. 12).

## Discussion

The qualitative characteristics revealed complex interplay among various agro-morphological traits in the 33 cassava varieties. Positive correlations were observed between several traits. This suggests that certain characteristics may influence or be influenced by one another. For instance, the association between color of apical leaves and the leaf color suggests that these traits may be genetically linked or affected by similar environmental factors. This aligns with findings that qualitative traits in plants are typically governed by a small set of genes, resulting in noticeable correlations between traits^21^. Another notable positive correlation is between the shape of the central leaf and branching/non-branching. This strong association suggests that the morphology of the central leaf could be a key factor in determining the plant’s branching pattern. Understanding these relationships is vital for comprehending plant architecture, which is shaped by both genetic and environmental influences^22^. The association between petiole color and the color of root cortex further supports that these traits may share a common developmental pathway, as suggested by previous studies on plant morphology^23^. Conversely, the negative correlations identified in the study provide insights into potential trade-offs or competitive relationships among traits. For instance, the negative correlation between the color of apical leaves and the shape of the central leaf suggests that the development of one trait in a specific direction may limit the development of the other. This aligns with the concept of phenotypic plasticity, where plants exhibit varying traits in response to environmental conditions, potentially leading to trade-offs in resource allocation^24^. Moreover, the negative relationship between leaf color and stem cortex color suggests that these traits may be inversely related. This could be due to competitive resource allocation during development^25^. The correlations involving root shape, external color of storage root, and petiole orientation suggest that root morphology may also be influenced by above-ground traits. This aligns with the understanding that plant morphology is interconnected^26^. This interconnectedness is critical for plant adaptation to varying environmental conditions, as morphological traits can influence physiological performance and overall fitness^27^.

PCA is an effective method for simplifying complex data by converting a large set of correlated variables into a smaller set of uncorrelated components that retain most of the important information. The PCA identified the traits which implied the greatest influence on variabilities observed in the 33 cassava varieties. Findings revealed significant insights into the relationships among these traits and their contributions to overall variation. The identification of two PCs is noteworthy, as it highlights the effectiveness of PCA in simplifying complex multivariate data while retaining essential information about trait interrelationships^28^. The PC1 is particularly influential with the color of apical leaves. This finding suggests that variations in leaf color are a primary factor in distinguishing between cassava varieties. It aligns with previous research indicating that leaf color reflects underlying physiological conditions and environmental adaptations^29^. The significant role of leaf color in PCA aligns with previous studies. These studies show that leaf pigmentation is altered by factors such as light availability and water supply. This further supports the relevance of leaf color in plant classification^30^. PC2 accounts for the shape of central leaf being a major contributor. This emphasizes the importance of leaf morphology in the overall assessment of cassava diversity. The positive association of leaf shape with other traits suggests that these characteristics may be genetically linked, which is important in breeding programs aimed to enhance specific traits^31^. The two components explain a large part of the data’s structure. However, they do not capture everything. Further analysis may be needed. Additional traits and their interactions could be explored further. The loadings of individual traits on the PCs revealed intricate relationships among the qualitative characteristics. In PC1, traits such as orientation of petiole and leaf color exhibit high positive loadings, indicating that varieties with specific petiole orientations and leaf colors tend to cluster together. This clustering suggests potential genetic or environmental influences on these traits, which can inform selection strategies in breeding programs^32^. Conversely, traits such as branching/non-branching and stem cortex color show negative loadings. These negative loadings indicate an inverse relationship with the positively loaded traits. This finding aligns with the concept of trade-offs in plant morphology, where certain traits may compete for resources during development^33^. In PC2, the shape of central leaf trait and the color of root cortex demonstrated the highest positive loadings, underscoring their significance in differentiating cassava varieties. The positive loadings of root shape and the external color of storage roots emphasize the importance of these root traits. They are key factors in evaluating the overall diversity of cassava varieties. This finding is especially valuable for breeding programs that focus on improving root quality and yield. Root morphology can significantly impact plant performance and adaptability^34^. The insights gained from this PCA are invaluable for cassava breeding programs. By understanding the relationships among qualitative traits, breeders can make informed decisions when selecting for desirable characteristics that enhance yield, disease resistance, and adaptability. The identification of traits with high loadings on the PCs can guide future research directions, particularly in exploring the genetic basis of these traits and their interactions with environmental factors^35^.

Hierarchical classification clustered the 33 varieties into 3 distinct groups. These groups were formed based on the similarities observed in the traits analyzed, highlighting the natural classification of the varieties according to their shared characteristics. This approach, which relies on the analysis of qualitative characteristics, provides an understanding of the diversity and genetic relationships among the varieties^36^. The genetic similarity observed among the cassava varieties for the 13 qualitative traits varied from 0 to 10, with the average similarity score being 5. This range indicates that some varieties share strong genetic similarities. However, others exhibit distinct differences in their traits. This suggests a moderate level of diversity within the group^37^. Clustering revealed that the cassava varieties formed three distinct groups using a similarity threshold of 4. Clusters I and III contained more varieties, while Cluster II had fewer. This distribution suggests that Cluster II may have a higher degree of variability or unique trait combinations compared to the larger clusters^38^. The grouping of varieties based on their qualitative traits highlights the potential for selecting varieties with desirable attributes. These selections can be used in breeding and cultivation programs. This approach may help improve the productivity and resilience of cassava crops^39^.

The variability observed among the 33 cassava varieties for qualitative traits provides valuable insights into their morphological diversity and genetic makeup. The color of apical leaves exhibited considerable variation. The median indicated that most varieties clustered around this midpoint. This wide distribution indicates a high level of diversity in apical leaf color. This trait may serve as an important marker for identifying and classifying different varieties^40^. The shape of the central leaf displayed a relatively balanced distribution, implying moderate variability in leaf shape, which could be linked to environmental adaptation or genetic differences^41^. In terms of petiole color, the mean indicated moderate variability. Range and median values suggested potential utility for selection in breeding programs targeting aesthetic or functional traits^42^. The leaf color trait reflected more uniformity compared to other traits. The consistency in this trait may highlight its stability under different growing conditions^43^. Variations in leaf vein color suggest that this trait may be useful for distinguishing varieties with distinct vascular patterns. These differences could potentially influence physiological functions, such as nutrient transport^44^. For the color of stem exterior a moderate variability was exhibited which indicates its utility in identifying visually distinct varieties^45^. The branching/non-branching trait exhibited a binary distribution suggesting its potential relevance for characterizing plant architecture and growth patterns^46^. The root shape reflected more uniformity, potentially influenced by breeding selection for specific root types preferred by farmers and consumers^47^. The external color of storage roots demonstrated moderate variability. Such differences in color could indicate varying levels of pigmentation, which might be associated with nutrient content or market preferences^48^. Similar patterns were noted for the color of the root cortex and the color of the flesh, both exhibiting low variances. This suggests a relatively uniform pigmentation for these traits, which may be attributed to specific genetic markers^49^. The orientation of the petiole showed moderate variability, with most varieties tending toward intermediate orientations. This could influence the canopy structure and light capture efficiency^50^. The color of stem cortex had distributions implying some consistency, which may be advantageous for selection purposes related to stem strength and pest resistance^51^.

The 33 cassava varieties exhibited significant variations across the 7 quantitative morphological traits. Findings highlighted considerable variability in the majority of traits, demonstrating the genetic diversity present within the cassava varieties. This variability suggests a high level of heterogeneity, making it a valuable resource for future breeding programs. These programs could focus on improving cassava traits such as yield, disease resistance, and adaptability to different growing conditions. The genetic diversity offers potential for selecting superior varieties with desirable traits, which could enhance the productivity and sustainability of cassava cultivation. Additionally, the number of leaf lobes showed moderate variability, with most varieties having a high number of lobes. This may be linked to improved photosynthetic efficiency and growth potential^52^. The levels of branching showed a relatively high standard deviation. The variation in branching levels could influence plant architecture, light interception, and yield potential^53^. Maturity displayed minimal variability. This indicates that most varieties matured within a narrow timeframe. Such uniformity is advantageous for synchronized harvesting and management^54^. For the number of tubers, the relatively low variability highlights the stability of this yield component, which is critical for selection in breeding programs^55^. The consistency in yield suggests the varieties have reliable productivity, which is essential for large-scale cultivation^56^. The starch content showed greater variability, with a wide range indicating genetic differences among varieties. This variability suggests potential applications for selecting varieties based on starch yield and quality^57^. The iron content had the highest variability. This variability highlights the potential for bio-fortification programs targeting iron-enriched cassava varieties to address micronutrient deficiencies^58^. The quantitative traits displayed varying degrees of variability, reflecting both genetic diversity and environmental influences. Traits such as maturity and the number of tubers showed stability. In contrast, starch and iron content exhibited wider ranges, providing opportunities for targeted breeding programs. These findings suggest the importance of quantitative traits in selecting cassava varieties for enhanced yield, nutritional value, and adaptability.

The correlation analysis of 7 quantitative traits in the 33 cassava varieties demonstrated both positive and negative associations among the evaluated characteristics. Positive correlations were observed between the number of leaf lobes and traits such as tuber number, yield, and starch content, suggesting that higher leaf lobe counts may enhance productivity. Branching levels showed a positive correlation with iron content, while maturity was positively associated with starch content. A strong positive relationship was also found between tuber number and yield, as well as yield and starch content, highlighting their interconnected influence on productivity and quality. Conversely, the number of leaf lobes showed negative correlations with branching levels and maturity, suggesting that an increased leaf lobe count may be linked to reduced branching and shorter maturity periods. The levels of branching displayed negative relationships with the number of tubers and starch content, while maturity negatively correlated with yield. Further negative associations were identified between the number of tubers and iron content, yield and iron content, and starch and iron content. These findings reveal complex trade-offs between yield, nutritional quality, and structural traits. They emphasize the importance of careful selection in breeding programs to balance productivity and plant characteristics effectively^59–61^. The PCA effectively captured the variability among the quantitative traits of the 33 cassava varieties, revealing that 2 PCs explained a total variation of 43.45%. The number of leaf lobes and the levels of branching were the primary contributors of the variances. These traits are critical for distinguishing cassava varieties and may serve as selection criteria for breeding programs^62^. Additional traits, such as maturity, the number of tubers, yield, and starch content, also contributed to the variation, indicating their importance in cassava evaluation. The relatively low contribution of iron content highlights the need for targeted improvement to enhance its nutritional value^63^.

The dendrogram revealed 5 distinct clusters among the cassava varieties, highlighting their genetic diversity and similarities based on quantitative traits. Cluster 1, the largest and most diverse group, included varieties such as COCI, KASALEKA, VULALIMA, and LOMAIVUNA, suggesting shared morphological and genetic characteristics. Cluster 2 grouped VULATOLU (Unbranched) with H-165, NAVOLAU, and others, indicating moderate similarity within this subgroup. A smaller, tightly knit Cluster 3, consisting of FALAWA, H-95, and H-97, suggests high genetic similarity among these varieties, potentially due to common parentage or breeding history^64^. Cluster 4, which includes NEW GUINEA, MERELESITA, and NOUMEA, demonstrated distinct traits, reflecting unique adaptations or breeding selection. The cluster 5 grouped multiple VULATOLU variations, including NADELEI and YASAWA VULATOLU, indicating genetic proximity and phenotypic resemblance within this subgroup.

## Conclusion

In conclusion, this study evaluated the genetic and morphological diversity of 33 cassava varieties in Fiji. Notable variations in qualitative traits such as leaf color, branching patterns, and root morphology were observed, reflecting genetic and environmental influences. Correlation analysis identified significant relationships between traits, such as the positive association between leaf lobes and productivity traits, which can inform breeding programs. PCA highlighted leaf morphology and branching as key traits contributing to variation, while dendrogram analysis classified the varieties into five clusters, offering insights into genetic similarity. The study highlights the importance of integrating morphological and genetic analyses to guide cassava breeding programs focused on improving yield, nutritional quality, and adaptability. The findings provide a solid foundation for developing enhanced cassava varieties that can address food security challenges and meet growing nutritional demands.

## Data Availability

The datasets used and/or analyzed during the current study are available from the corresponding author upon reasonable request.
